# Application of problem based learning (PBL) and case based learning (CBL) in the teaching of international classification of diseases encoding

**DOI:** 10.1038/s41598-023-42175-1

**Published:** 2023-09-14

**Authors:** Wanjun Yang, Hui Li, Aonan Su, Liping Ding

**Affiliations:** Department of Medical Records Statistics, Zhejiang Provincial People’s Hospital/People’s Hospital of Hangzhou Medical College, 158 Shangtang Road, Gongshu District, Hangzhou, 310000 Zhejiang China

**Keywords:** Health occupations, Health policy, Health services, Patient education, Public health

## Abstract

To study the application of PBL combined with CBL teaching mode in the teaching of international classification of diseases (ICD) encoding, so as to improve students' grasp of ICD encoding knowledge. From March 2020 to June 2020, 50 students majoring in health information management who were interns in a Grade A general hospital were selected as the research participants and randomly divided into experimental group and control group, 25 in each group. The experimental group and the control group adopted the PBL combined CBL teaching mode and the traditional teaching mode respectively to carry out the classification and coding teaching. The academic achievement of the two groups of students was evaluated by means of achievement assessment and questionnaire survey based on information processing theory. The scores of theoretical knowledge in the experimental group and the control group were 79.78 ± 8.55 and 70.92 ± 10.81, respectively, and the scores of skill operation in the two groups were 79.76 ± 8.28 and 70.00 ± 10.41, respectively. The test scores of the experimental group were higher than those of the control group, and the difference was statistically significant (*P* < 0.05). The scores of knowledge acquisition ability of experimental group and control group were 16.72 ± 1.79 and 16.60 ± 2.36, the scores of knowledge sharing ability were 24.20 ± 2.61 and 21.00 ± 2.65, the scores of knowledge storage ability were 20.80 ± 2.47 and 17.24 ± 4.90, respectively. The scores of knowledge application ability were 14.00 ± 1.80 and 11.00 ± 2.69, the scores of knowledge innovation ability were 20.16 ± 2.34 and 18.08 ± 3.70, and the total scores were 95.88 ± 6.08 and 83.92 ± 11.30, respectively. The scores of all questionnaires in the experimental group were higher than those in the control group. The scores of knowledge sharing ability, knowledge storage ability, knowledge application ability, knowledge innovation ability and total score between the two groups were higher than those of the control group, and the differences were statistically significant (*P* < 0.05). PBL combined with CBL teaching model has good academic achievement in ICD encoding teaching, which can significantly improve academic performance and learning ability, and is worthy of promotion.

## Introduction

Classification of diseases is to arrange and group diseases according to their etiology, anatomical location, clinical manifestations and pathological characteristics, so that they become an orderly combination^1^. The International Classification of Diseases (ICD) is a system that describes and codes mortality and morbidity events and is implemented by most World Health Organization (WHO) member States^2^. Accuracy of the ICD encoding is the cornerstone of successful health information efforts, and ICD is widely used by health care providers, hospitals, health care payers and governments to track health trends and statistics at the global, national and local levels, and to provide a reimbursement framework for health care based on disease diagnosis and severity^3^. Accurate coding is critical for clinical practice and research, billing and reimbursement, health service planning, and infrastructure development^4–6^. In addition, Zou et al.^7^ found that diagnosis-related groups (DRGs) were widely popularized and used in our country. Therefore, the requirement for ICD encoding was further improved, and the ICD encoding was gradually developed in the direction of specialization. However, behind this professional development, there is a big hidden danger, that is, the teaching of ICD encoding cannot keep up with the rapid development of the case information management industry, and there is a lack of practical teaching materials for ICD encoding to ensure the accuracy and efficiency of teaching^8^. Therefore, how to reform the teaching method of ICD encoding, cultivate students' logical thinking in coding, improve students' coding skills, and lead students to obtain better learning results in a limited time has become an urgent problem to be solved.

At present, the teaching of ICD encoding in China mostly adopts the traditional teaching mode, that is, Lecture Based Learning (LBL), which adopts an “indoctrination” teaching method, taking teachers as the teaching center, students passively accepting knowledge, and lacking self-reflection and learning ability cultivation of students. In order to make up for the shortcomings of LBL teaching mode, medical teaching reform has developed a variety of teaching modes, among which Problem Based Learning (PBL) and Case Based Learning (CBL) are the two teaching modes that are currently more widely used. However, when used alone, both PBL and CBL have limitations^9–11^. CBL requires teachers to invest significant time in preparation to prepare a sufficient number of cases to support clinical teaching. At the same time, CBL also requires teachers to create a set of questions for students to discuss, resulting in a lack of initiative and general enthusiasm for the learning experience. In contrast, PBL places the student at the center and in the driver's seat of the classroom process. This function requires them to spend a lot of time preparing questions and materials before class, which is extremely difficult for medical students due to their heavy coursework. In addition, PBL emphasizes students' subjective initiative too much; However, the lack of guidance from teachers can cause students to miss the focus of the course, thus affecting the overall quality of the course.

The PBL combined with CBL teaching mode combines the advantages of CBL and PBL, problem-centered, case-guided, and the teaching teacher selects typical clinical cases in practical work as teaching cases, and raises questions to guide students to organically combine theoretical knowledge with coding practical problems, and a number of studies have found that this model has obvious advantages over traditional LBL^12–15^. Under the guidance of teachers' overall planning, the teaching method of PBL-CBL gives full play to the vitality and attraction of actual cases by simulating real application scenarios in actual cases, thus effectively improving the clinical practice and solving practical problems of medical students. At the same time, the problem-oriented approach emphasizes the divergent thinking of students' subjective initiative in learning and guides students to solve problems through self-study, interaction, discussion and group cooperation, which can increase students' learning interest and sense of participation and cultivate students' independent learning ability. However, at present, the teaching model of PBL combined with CBL is rarely applied to the teaching of ICD encoding, and there is a lack of systematic and scientific academic achievement evaluation system^12–15^.

Therefore, based on the understanding of the above problems, this study integrates the teaching design of ICD encoding, introduces the teaching model of PBL and CBL into the teaching process, and establishes a standardized teaching case bank, standardized teaching process and a scientific teaching evaluation system based on information processing theory. Information processing theory, also known as modern cognitive theory, holds that the process of information processing is the process of information acquisition, input, storage, extraction and application, and the process of personal knowledge management is also the process of knowledge acquisition, storage, application and sharing by individuals, which is widely used in human resources, medical care, clinical diagnosis and treatment, education system and other fields, and good results have been achieved^16^. The students' learning of the ICD encoding courses are also the process of acquiring, entering, storing, extracting and applying knowledge. Based on the information processing theory, this study constructs a scientific academic achievement evaluation system: a student academic achievement evaluation system that includes five core abilities: knowledge acquisition ability, knowledge sharing ability, knowledge storage ability, knowledge application ability and knowledge innovation ability. This research is summarized and reported as follows.

## Materials and methods

### Participants

This study selected 50 students majoring in health information management who were interns in Zhejiang Provincial People's Hospital as the research participants. In a simple random way, they were divided into experimental group and control group. The experimental group adopted the teaching mode of PBL combined with CBL for ICD encoding, and the control group adopted the traditional teaching mode for ICD encoding, with 25 students in each group. Randomization is the use of special methods that use the equal probability of something happening to each individual in the population or sample. Neither the student nor the teacher knew the group information before the class started (double blind). In this study, a simple random grouping was carried out by drawing lots. 50 subjects were numbered according to the natural numbers 1–50, and the numbers were written and folded on strips of paper. After folding, the strips were placed in an opaque container and stirred evenly. 50 students took one piece of paper at a time, and drew 50 times without putting it back. If the number drawn is odd, it goes to the PBL-CBL group, if the number drawn is even, it goes to the LBL group. Arrange to come to the hospital to complete the internship course of ICD encoding twice a week, with 2 class hours each time and 40 min each class hour. This study was conducted in accordance with the guiding principles of the Declaration of Helsinki and approved by the Ethics Committee of Zhejiang Provincial People's Hospital. Written informed consent was obtained from all students.

### Methods

**Figure 1:**
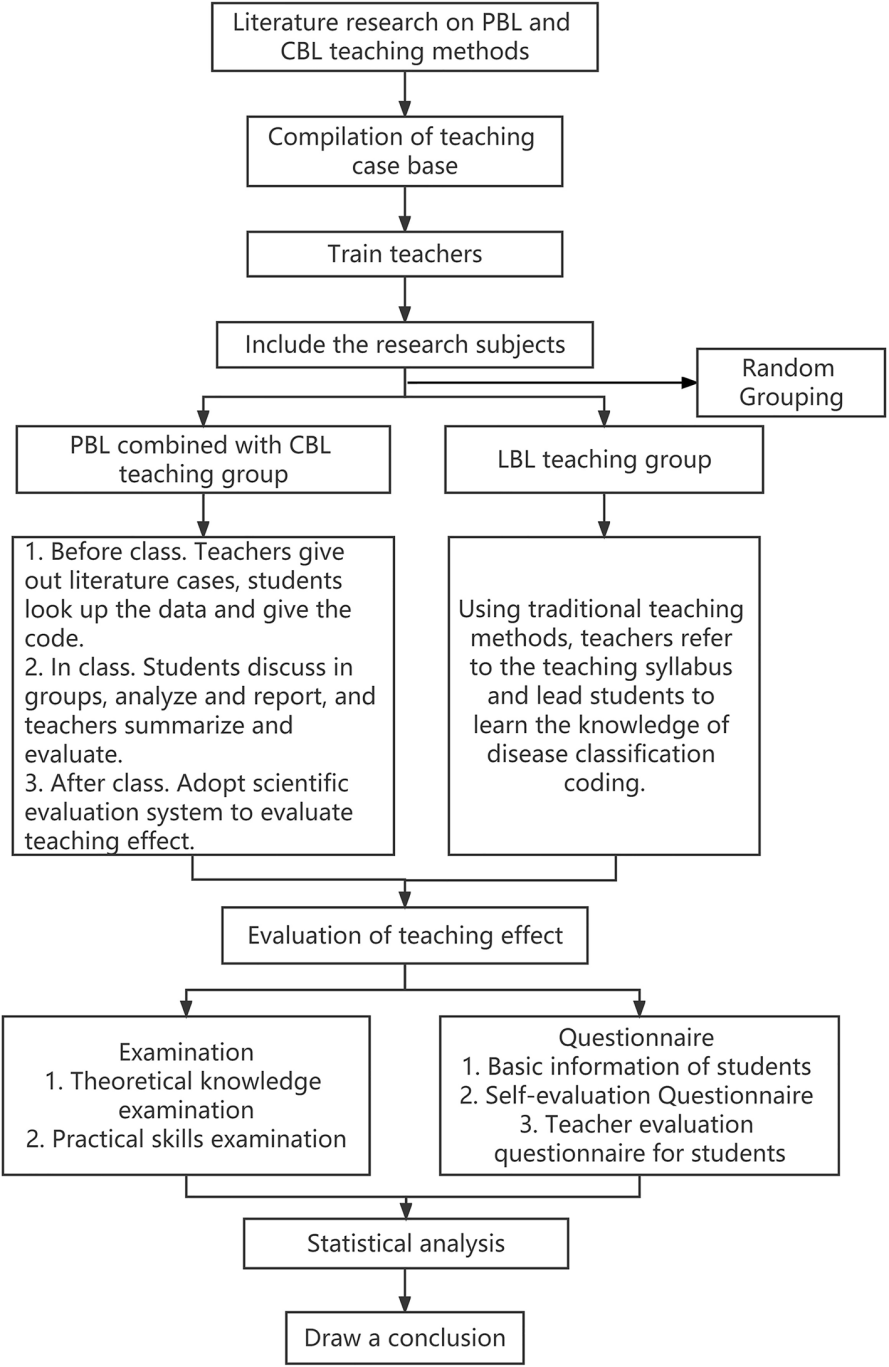
Project implementation flow chart.

The project implementation flow chart of this study is shown in Fig. 1. Before the project began, relevant literature of PBL and CBL teaching methods was studied firstly, and then standardized teaching case base suitable for PBL combined with CBL teaching model was prepared and teachers were trained. At the beginning of the project, the subjects were randomly divided into two groups, PBL-CBL method and LBL method were used respectively. After the end of the project, the academic achievement was evaluated, and the obtained data was statistically analyzed, and finally the conclusion was drawn.

This study adopts the ADDIE model to make the teaching plan. The ADDIE model includes five steps, namely Analysis, Design, Development, Implementation and Evaluation: (1) The first step is analysis, which identifies the objectives, audiences and needs of the course and training. In this step, the teacher identifies where the student is coming from, the professional foundation, what still needs to be learned and mastered, what the teacher needs to teach, and what the student's learning style is. By answering these questions, teachers can determine that the core content and goal of the course and training program is to establish a set of standardized teaching case base and standardized teaching process of PBL and CBL teaching mode suitable for the ICD encoding, so as to improve students' grasp of the knowledge of ICD encoding. (2) The second step is design, which ensures the effectiveness and feasibility of the curriculum and training program. In this step, the teacher confirms the content and tasks of the study teaching plan, the teaching methods used, how to evaluate learning outcomes, etc. By answering these questions, the project has designed an effective curriculum and training program. (3) The third step is development, which translates the curriculum and training programs into actual teaching materials. In this step, the teacher integrated the contents constructed in the design stage, formulated the case base and teaching plan of disease classification code, standardized teaching process of PBL-CBL group and LBL group, and prepared and improved teaching effect evaluation tools. Through this work, teachers can develop a complete ICD encoding curriculum and training program. (4) The fourth step is implementation, which is to actually apply the curriculum and training program to real scenarios. In this step, the teacher arranges the course and training time, determines the teaching venue and equipment, assigns teachers, teacher training, etc. The training includes all the training courses, learning results, implementation methods and learning effect evaluation. In this stage, the teacher can apply the curriculum and training programs to practical scenarios to achieve the desired results. (5) The fifth step is evaluation, which assesses the effectiveness and feasibility of ICD encoding courses and training programmes. In this step, teachers use test and assessment methods of achievement assessment and questionnaire surveys to measure learning outcomes and collect feedback and opinions from students to make improvements to the curriculum and training programs. Through these efforts, course participants can evaluate the effectiveness of ICD encoding courses and training programs and improve them.

#### Compile teaching case library

For the 22 chapters of ICD-10, the backbone of ICD encoding business was organized to collect, integrate and summarize typical clinical cases, and a standardized teaching case base was made. After the preparation, the case writing team had a collective discussion, repeated modification, and completed the design and preparation of the case.

The teacher divides each case into 3–4 acts according to the actual situation of clinical records, and gives different information in each act to guide students to discuss and analyze.

Take ICD-10 coding analysis of cerebral infarction as an example. In this case, a 69-year-old male patient was admitted to hospital with "aphasia and right limb weakness for 3 days". Head Magnetic Resonance Imaging (MRI) showed multiple small flaky, blotchy and slightly longer T1 and slightly longer T2 signals in the left basal ganglia, radiating crown and center of the hemioval. Diffusion Weighted Imaging (DWI) showed a high signal with limited diffusion, while Apparent Diffusion Coefficient (ADC) showed a low signal. Diagnosis: Multiple cerebral infarction (acute stage) in the left basal ganglia, radial crown and center of hemioval. Digital subtraction angiography (DSA) showed that the initial part of the right internal carotid artery was about 95% stenosis, the initial part of the left internal carotid artery was blocked, the opening of the right vertebral artery was 95% stenosis, and the residual cerebral atherosclerosis changed. Discharge diagnosis: (1) cerebral infarction; (2) Atherosclerosis (left internal carotid artery occlusion, severe stenosis of right internal carotid artery initiation and right vertebral artery).

In the first act, Xiao Zhang (a pseudonym) went to the provincial People's Hospital for treatment due to "aphasia and right limb weakness for 3 days". The receiving doctor conducted an interrogation, visual examination and auscultation examination on the patient, and gave the diagnostic opinion of "multiple cerebral infarction (acute stage) in the left basal ganglia, radial coronal and semi-oval center" according to the results of head MRI and routine examination. In this scene, the MRI and routine examination results of the patient's head were presented for students' analysis. In the second act, the results of Xiao Zhang's DSA test are available, and the results are presented to the students for discussion. In the third act, Xiao Zhang is discharged from the hospital, and the doctor gives the following discharge diagnoses: (1) cerebral infarction; (2) Atherosclerosis (left internal carotid artery occlusion, severe stenosis of right internal carotid artery initiation and right vertebral artery). Students discuss and analyze the final discharge diagnosis. In this case, the main discussion directions are: (1) clarifying the staging diagnosis of cerebral infarction, (2) judging the responsible vessels of cerebral infarction, (3) determining the etiological classification of cerebral infarction, and (4) giving the corresponding ICD-10 code.

The experts who developed the case are all experts who have been engaged in ICD coding for more than 10 years and have rich knowledge accumulation and practical experience, including 4 professors and associate professors and 6 coding teachers.

#### Train teachers

Organize teachers to study and discuss PBL and CBL teaching models together, conduct pre-trial lectures according to the written teaching case base, improve teaching methods according to the effects of trial lectures, and timely communicate and share with other teachers. So that the teacher's teaching concept gradually changed from the traditional indoctrination teaching to discussion teaching, from "teaching" to "teaching—guidance".

#### Teaching methods

**Control group:** The traditional teaching method was adopted. The teachers were instructed to refer to the teaching syllabus and the students were instructed to learn the relevant knowledge of ICD encoding.

**Experimental group:** PBL combined with CBL teaching mode was adopted, typical clinical cases were selected, and questions were designed in the medical records based on the requirements of the teaching syllabus. 25 students were divided into 5 groups, and the analysis of each medical record was required to be completed by group cooperation. Specific process: (1) Before class. Teachers: Publish the books and literature that need to be read in advance, as well as the teaching cases for the next lesson (emphasis on case-guided CBL), and ask the questions to be solved for this disease classification coding course (emphasis on problem-centered PBL). Students: Consult the information on their own and give the disease code. (2) In class. Grouping of students: 5–6 people in a study group, select the chairman and clerk; Case analysis: The prepared cases are analyzed and problem-centered discussion is carried out. The main discussion directions are disease etiology, disease diagnosis and disease coding. Finally, the optimal choice of ICD encoding is given after summary and analysis. Case discussion summary: At last, the case discussion is summarized, including the student summary (student groups report in PPT or Word form) and the teacher summary (teacher gives the correct answer and analysis). (3) After class. Teachers publish the thinking and discussion questions in this section to guide students to learn independently. At the end of the course, a scientific evaluation system is adopted to evaluate the academic achievement of PBL combined with CBL.

In order to control the pollution in the educational experiment, in our study, the experimental group and the control group adopted the PBL-CBL teaching method and the LBL teaching method respectively. The classrooms for the classes and examinations of the two teaching methods were separated, and the courses were carried out simultaneously, and it was impossible to communicate with each other, so the control group could not access the content provided by the experimental group.

#### Academic achievement evaluation index

(1) Performance assessment: Organize students to conduct assessment of theoretical knowledge and skill operation, and record test results. In this study, the theoretical knowledge and skill operation assessment were carried out in the form of off-line closed-book written tests. The theoretical knowledge examination was in the form of single-choice questions, multiple-choice questions and judgment questions, and the examination content was the professional knowledge necessary for ICD encoding, including the basic knowledge of the International Classification of Diseases, anatomy, and the selection of major diagnoses. The assessment form of skill operation assessment is question and answer, that is, the diagnosis and treatment process of clinical cases is given, students are asked to analyze the cases, choose the main diagnosis and main operation, and give the corresponding code, and finally the teacher grades the paper.

(2) Questionnaire survey: Before and after the implementation of PBL and CBL teaching model, students were surveyed by self-filling questionnaire. This study designs questionnaires based on information processing theory, literature research and expert interview guidance. According to this theory, human information processing is a process of information acquisition, input, storage, extraction and application^16^. Based on information processing theory, literature research and expert interview, This study divides the questionnaire into five dimensions: knowledge acquisition ability, knowledge sharing ability, knowledge storage ability, knowledge application ability and knowledge innovation ability. Each dimension is measured with 6–7 questions, and the score is divided into 5 scales according to degree, from 1(not at all) to 5(always). In this study, Cronbach's α was used to evaluate the reliability of the questionnaire with 30 students as the sample. Cronbach's α in each sub-dimension was acceptable (α = 0.60–0.84). The validity of the questionnaire was evaluated by group discussion, and the questionnaire was modified according to the opinions of experts. The validity of the questionnaire was confirmed to be good. The questionnaire includes three parts: students' basic information, students' self-assessment questionnaire and teachers' evaluation of students. Based on information processing theory, the survey mainly includes five dimensions: students' knowledge acquisition ability, knowledge sharing ability, knowledge storage ability, knowledge application ability and knowledge innovation ability.

Knowledge acquisition ability refers to people's ability to acquire knowledge through external sensing, communication and mass media, as well as through consulting, information retrieval, reading books and documents. Information acquisition ability is the most basic ability that people can use information.

Knowledge sharing ability refers to the ability of people to share and exchange knowledge with others, so that knowledge can spread from personal experience to the level of the organization. In this way, within the group, students can obtain solutions to problems by querying the organization's shared knowledge, so as to improve learning efficiency.

Knowledge storage ability refers to people's ability to internalize the acquired knowledge and received information into their own knowledge, so as to provide reserves for future knowledge application.

Knowledge application ability means that after acquiring and interpreting the information provided by the test questions, according to the setting of the test questions, people can quickly, accurately and completely select the appropriate knowledge from the existing knowledge reserves in a targeted way, organize and use the stored knowledge in a hierarchical and clear way according to certain logical relations, and freely invoke the relevant knowledge to analyze and solve problems.

Knowledge innovation ability refers to the ability to improve or create new knowledge or things to meet certain needs in a specific environment by using existing knowledge and materials to put forward ideas that are different from the conventional or ordinary people's thoughts.

#### Statistical analysis

In this study, epidata3.1 was used for double data entry and SPSS 22.0 software was used for statistical analysis. Measurement data are expressed as ${\overline{\text{x}}}$ ± S, and counting data is expressed as n (%). For measurement data, Shapiro–Wilk test was used for normality test, and *t*-test was used for comparison between groups if normal distribution was met. The Mann–Whitney *U* test was used for comparison between groups if the distribution did not conform to normal. Statistical data were compared between groups using chi-square test. Two-sided test was used for all analyses, and the test level was α = 0.05.

## Results

### Basic information description

A total of 50 questionnaires were distributed in this study, with 25 in the experimental group and 25 in the control group respectively. 50 effective questionnaires were collected with effective recovery of 100%. There were 26 males and 24 females, and there was no significant difference between the experimental group and the control group (P = 0.571). The mean age of the experimental group was 20.80 ± 1.35 years old, and that of the control group was 21.12 ± 1.59 years old. There was no significant difference in age between the two groups (P = 0.447) (shown in Table 1).

**Table 1:** Comparison of general data between the experimental group and the control group.

Variable	Experimental group (n = 25)		Control group (n = 25)		*t/χ*^2^	*P*
	Number	Constituent ratio (%)	Number	Constituent ratio (%)		
Gender						
Male	12	48.0	14	56.0	0.321	0.571
Female	13	52.0	11	44.0		
Age (years, ${\overline{\text{x}}}$ ± S)	20.80 ± 1.35		21.12 ± 1.59		0.766	0.447

### Evaluation of academic achievement of two teaching modes

#### Examination scores of experimental group and control group

The theoretical knowledge scores of the experimental group and the control group were 79.78 ± 8.55 and 70.92 ± 10.81 points, and the skill operation scores of the two groups were 79.76 ± 8.28 and 70.00 ± 10.41 points, respectively. The examination scores of the experimental group were higher than those of the control group (shown in Table 2).

**Table 2:** Comparison of examination scores between experimental group and control group.

Category	Experimental group (n = 25)	Control group (n = 25)	*t*	*P*
Theoretical knowledge scores	79.78 ± 8.55	70.92 ± 10.81	3.215	0.002
Skill operation scores	79.76 ± 8.28	70.00 ± 10.41	3.669	0.001

#### Questionnaire scores of the experimental group and the control group

The scores of knowledge acquisition ability of the experimental group and the control group were 16.72 ± 1.79 and 16.60 ± 2.36 points, the scores of knowledge sharing ability of the two groups were 24.20 ± 2.61 and 21.00 ± 2.65 points, and the scores of knowledge storage ability of the two groups were 20.80 ± 2.47 and 17.24 ± 4.90 points, respectively. The scores of knowledge application ability of the two groups were 14.00 ± 1.80 and 11.00 ± 2.69 points, and the scores of knowledge innovation ability of the two groups were 20.16 ± 2.34 and 18.08 ± 3.70 points, respectively. The total scores of the two groups were 95.88 ± 6.08 and 83.92 ± 11.30 points, respectively (shown in Table 3).

**Table 3:** Comparison of questionnaire scores between experimental group and control group.

Category	Experimental group (n = 25)	Control group (n = 25)	Test statistics	P
knowledge acquisition ability scores	16.72 ± 1.79	16.60 ± 2.36	342.000	0.560
Knowledge sharing ability scores	24.20 ± 2.61	21.00 ± 2.65	− 4.302	< 0.001
Knowledge storage capacity scores	20.80 ± 2.47	17.24 ± 4.90	471.500	0.002
Knowledge application ability scores	14.00 ± 1.80	11.00 ± 2.69	506.000	< 0.001
Knowledge innovation ability scores	20.16 ± 2.34	18.08 ± 3.70	− 2.378	0.021
Total scores	95.88 ± 6.08	83.92 ± 11.30	515.000	< 0.001

#### Normality test of test scores and questionnaire scores of experimental group and control group

Shapiro–Wilk normality test was used in this study. According to the results of normality test, the scores of theoretical knowledge, skill manipulation, knowledge sharing ability and knowledge innovation ability were consistent with the normal distribution (*p* > 0.05). Knowledge acquisition ability, knowledge storage ability, knowledge application ability and total score do not accord with normal distribution (*p* > 0.05) (shown in Table 4).

**Table 4:** S–W normality test results of examination scores and questionnaire scores of experimental group and control group.

Method	Category	*W* value	*P*
Examination	Theoretical knowledge scores	0.997	0.423
	Skill operation scores	0.971	0.248
Questionnaire survey	knowledge acquisition ability scores	0.951	0.038
	Knowledge sharing ability scores	0.982	0.647
	Knowledge storage capacity scores	0.924	0.003
	Knowledge application ability scores	0.913	0.001
	Knowledge innovation ability scores	0.961	0.099
	Total scores	0.930	0.006

#### Comparison of examination scores between the experimental group and the control group

According to the results of normality test, the scores of theoretical knowledge and skill operation conform to the normal distribution, so *t* test was used to compare the test scores of the experimental group and the control group. The results showed that the scores of theoretical knowledge and skill operation in experimental group were higher than those in control group, and the difference was statistically significant (*P* < 0.05) (shown in Table 2).

#### Comparison of questionnaire scores between experimental group and control group

According to the results of normality test, knowledge sharing ability and knowledge innovation ability conform to the normal distribution, and *t* test was used to compare the differences between the experimental group and the control group. Knowledge acquisition ability, knowledge storage ability, knowledge application ability and total score did not conform to the normal distribution, and the Mann–Whitney *U* test was used to compare the differences between the two groups. The analysis results showed that knowledge sharing ability, knowledge storage ability, knowledge application ability, knowledge innovation ability, total score and total score of experimental group were higher than those of control group, and the difference was statistically significant (*P* < 0.05) (shown in Table 3).

## Discussion

PBL and CBL teaching models were first proposed by Canada's Kmaster University in 1969^17,18^. Later, PBL and CBL teaching models were popularized and applied in medical colleges in the United States, Europe, Britain and other countries^19,20^. In the late 1980s, some medical colleges in our country began to try PBL and CBL teaching model^21^. After years of development, PBL and CBL teaching models have been widely used in medical education^9,22^. In a Japanese study, education using PBL improved the skills and communication skills students needed to solve social problems^23^. Li et al.^24^ believe that competency-oriented combined with CBL teaching method is an effective way to improve students' professional knowledge, increase their language expression ability, and enhance their interpersonal relationship and teamwork ability, which is worthy of promotion in medical teaching. However, Wang et al.^25^ found that PBL and CBL have different emphases, but the combination of PBL and CBL can improve the academic achievement of oral education: PBL teaching significantly improves the independent learning ability of students in theory courses; CBL helps students diagnose and develop treatment plans for actual cases during the internship.

However, in the teaching of ICD encoding, PBL combined with CBL teaching mode is less applied. In addition, the current PBL combined with CBL teaching model lacks a scientific and systematic evaluation system in the evaluation of academic achievement. It only relies on theoretical assessment results or self-made questionnaires to evaluate academic achievement^12–15^, which is simple in content and single in form, and lacks theoretical guidance.

Information processing theory, also known as modern cognitive theory, was produced in the mid-1950s. According to this theory, people's information processing is the process of information acquisition, input, storage, extraction and application, and the process of personal knowledge management is also the process of knowledge acquisition, storage, application and sharing. It has been widely applied in human resources, medical care, clinical diagnosis and treatment, education system and other fields, achieving good results^12^. Based on the social information processing theory, Wang et al.^26^ provided a new theoretical explanation for how and when leadership humor affects subordinates' cross-border behavior. Gordon et al.^27^ combined information processing methods with embodied cognition theory to propose a new cognitive model that can help children learn and solve math problems. Albanese et al.^28^ concluded that information processing theory provided better theoretical support for PBL than contextual learning theory after comparing multiple theories. Noordegraaf-Eelens et al.^29^ believe that problem solving ability in real life involves cognitive process, while the existing PBL model enhances the attention to knowledge acquisition of specific problems, but separates education from the whole world, lacking the whole process of knowledge cognition.

Therefore, based on the understanding of the above problems, this study introduces the teaching mode of PBL and CBL into the teaching process. Based on the information processing theory, a academic achievement evaluation system for students is established, which includes five core abilities: knowledge acquisition ability, knowledge sharing ability, knowledge storage ability, knowledge application ability and knowledge innovation ability.

Our study found that the theoretical knowledge and skill operation scores of PBL-CBL group were higher than those of CBL group, and it could improve students' knowledge sharing ability, knowledge storage ability, knowledge application ability and knowledge innovation ability, but there was little difference in knowledge acquisition ability between the two groups. This is similar to previous findings^9–13^. Zhao et al.^12^ randomly assigned fourth-year clinical medicine students and residents to the PBL combined CBL group and the LBL group. The research results showed that compared with the LBL teaching model, the PBL combined CBL teaching model was more conducive to improving the clinical skills of medical students and residents. A study by Sangam et al.^11^ introduced innovative teaching methods such as CBL and PBL, and the results showed that CBL as an anatomy teaching method is a more effective way to improve and retain knowledge. In nursing practice teaching, research shows that PBL combined with CBL teaching model can stimulate students' interest in learning, improve self-learning motivation, improve students' theoretical and operational performance, so as to help students better understand patients, experience various clinical scenarios, and promote the improvement of clinical nursing ability^13^. Shen et al.^14^ found that compared with traditional teaching methods, the teaching method combined with CBL and PBL can improve medical students' self-perception ability, after-class test scores and clinical practice ability, improve students' satisfaction with the course, and help increase the enrollment of medical students in neurosurgery. Liu et al.^15^ showed that PBL-CBL method was superior to the traditional teacher-centered method in academic knowledge acquisition, case analysis ability and student satisfaction in the teaching of maxillary sinus floor lifting. Possible reasons are as follows: Compared with traditional teaching mode, PBL combined with CBL teaching mode enables students to get in-depth thinking in a short time, make efficient use of limited class hours, improve students' interest in ICD encoding and improve their comprehensive ability on the basis of ensuring teaching quality and effect, so that students can really learn something in the study of ICD encoding. However, in this study, there was little difference in knowledge acquisition ability between the experimental group and the control group, and the results of previous studies were inconsistent^9,13^. Liu et al.^15^ showed that in the teaching of maxillary sinus floor lifting, PBL-CBL method was superior to the traditional teacher-centered method in academic knowledge acquisition ability. Wang et al.^25^ showed that there was no statistically significant difference in the ability to collect data between the two groups after the PBL course. Knowledge acquisition is mostly a process in which students collect information related to the course by themselves before the course starts, and the motivation for knowledge acquisition comes from the individual or organization's own demand for knowledge^9^. The small difference between the two groups of this index indicates that the group randomization level is high and the comparability is strong. Also from the reverse, it is precisely because the two groups implemented different teaching modes that the test results and multiple learning abilities of the experimental group were superior to those of the control group.

There was a major concern in this study that the effects of student-centered teaching methods compared to teacher-centered teaching methods have been demonstrated in the literature. This study raises some thoughts for education stakeholders: (1) For universities, hospitals and other education sectors: The important goal of the curriculum is the mastery and application of knowledge, and a number of studies^12–15^ show that compared with the teacher-centered teaching method, the student-centered teaching method has a better effect, which helps the education department to improve the teaching quality, attract students, and provide references for the reform and innovation of the education department. (2) For teachers: It is helpful to improve teacher performance assessment results: good classroom teaching effect, high student satisfaction, and strong curriculum innovation, which are important items of teacher performance assessment; And it can reduce the burden of teachers in class: students are the main role in the classroom, teachers only need to guide and cooperate with students; In addition, it can increase the sense of professional identity and professional gain, so that teachers love their work more. (3) For students: the main body of the class will be changed from "teacher" to "student", increase students' sense of participation, add interest to the class, fully mobilize students' enthusiasm for learning, and cultivate students' ability to find and solve practical problems.

This study has some advantages. First, this study aims at improving the academic achievement of ICD encoding, providing more evidence for hospital teaching and the management of hospital medical record statistics department. Secondly, at present, the teaching mode of ICD encoding is mostly LBL, and the teaching mode of PBL combined with CBL has obvious advantages over the traditional LBL. To the best of our knowledge, this study is the first to apply the PBL combined CBL teaching model to the teaching of ICD encoding. Finally, we use the scientific theory guide—information processing theory for academic achievement evaluation, credibility is strong.

## Conclusions

The teaching model of PBL combined with CBL is better than that of traditional teaching methods in the teaching of disease classification and coding, showing higher scores, better knowledge sharing ability, knowledge storage ability, knowledge application ability and knowledge innovation ability. This paper shows that PBL-CBL is a promising new ICD encoding teaching model. On the one hand, it can increase students' sense of participation, make the class more interesting, fully mobilize students' learning enthusiasm, and cultivate students' ability to find and solve practical problems. On the other hand, good classroom teaching effect, high student satisfaction and strong curriculum innovation provide more basis for future hospital teaching and management of hospital medical record statistics department, and also provide experience for other educational institutions, which is worth promoting.

## Data Availability

The datasets used and/or analysed during the current study available from the corresponding author on reasonable request.
